# Alleviation of nephropathy during aging as complications of diabetic by natural products

**DOI:** 10.4314/ahs.v24i4.49

**Published:** 2024-12

**Authors:** Rami M Mosaoa, Taha A Kumosani, Soonham S Yaghmoor, Said S Moselhy

**Affiliations:** 1 Department of Biochemistry, Faculty of Science, King Abdulaziz University. Jeddah, Saudi Arabia; 2 Experimental Biochemistry Unit, King Fahd Medical Research Centre, King Abdulaziz University, Saudi Arabia; 3 Production of Bio-products for Industrial Applications Research Group, King Abdulaziz University, Jeddah, Saudi Arabia; 4 Department of Biochemistry, Faculty of Science Ain Shams University, Cairo, Egypt

**Keywords:** Aging, Diabetic nephropathy, AGEs, Inflammation, anserine, Vitamin E, rat

## Abstract

**Background:**

The most complications of chronic diseases as diabetic during aging is micro and microvascular disorders. Consumption of functional foods is very important in protection from these complications. We studied the impact of dipeptide anserine in combination with vitamin E in prevention diabetic nephropathy in diabetic rats.

**Methods:**

The study included 60 male albino rats sorted into five groups: GP (I): control and the other rat groups were induced diabetic by a single dose of streptozocine i.p, at dose of (55 mg/kg/b.w).GP II was considered as diabetic untreated. The other diabetic groups were treated with anserine (1mg/kg b.w, i.p), α-tocopherol (50, 00 IU/kg b.w) or combined. After 12 weeks, fasting serum was subjected for assay of glucose, glycated hemoglobin (HA1c), advanced glycated end products (AGEs), oxidative stress markers (MDA, SOD) and inflammatory markers (TNF-α and IL-6).

**Results:**

Data obtained revealed that, diabetic rats treated with anserine or α-tocopherol or combination improve abnormalities, glucose, HA1c, antioxidant enzymes, inflammatory mediators and AGEs versus untreated diabetic.

**Conclusion:**

Dietary supplement of natural products as anserine and α-tocopherol protect against micro and microvascular system, which is suggestive as alternative or complementary therapeutic agents for diabetic complications as neuropathy, nephropathy, and CVD.

## Introduction

The aging was associated with decline in physiological pathway efficiency as intolerance with high blood glucose. As a consequence, free glucose bind non enzymatically with amino group of different tissue proteins and causes denaturation and non functional that affecting different tissues. Diabetes mellitus (DM) is a chronic, severe condition characterized by persistently high blood glucose level, affecting various body functions^1^. The widespread and rapid prevalence of this disease control a substantial burden of mortality, morbidity, and healthcare^2^. Diabetes complications including micro or macro vascular disorders, such as nephropathy, retinopathy, and neuropathy^3^. The prevention and treatment of diabetic nephropathy (DN) in early stages is critical to avoid end stage renal disase. Lifestyle, feeding habits and physical activity are the main precautions of diabetes management and the prevention of late-stage diabetic complications^4^. Additionally, management and maintenance of blood glucose level is the first line for prevention its complications. Although various hypoglycemic agents as metformin or insulin are generally effective in controlling blood glucose, they are associated with numerous side effects and limited action in preventing or delay diabetic complications^5^. Peptides produced from animal or plant sources by enzymatic hydrolysis exert different biological activities as antioxidant, immunomodulatory, antiglycation and antitumor effects. Therapeutic natural peptides from animal or plant sources are considered as a safe, without side effects, it exerts potentially agent in many diseases^6^. Carnosine and anserine are derivatives from histidine amino acids and served as biologically buffer in tissue during cell cycle and metabolism. Carnosine showed free radical scavenging agent and antioxidant^7^. Biological radical scavenger agents including enzymes, peptides, proteins, and vitamins. Vitamins A, E and C are powerful antioxidant against free radical generation and peroxidation. The most effective one is Vitamin E (tocopherol) in support immune response, cell membrane integrity^8^-^10^. The most complications of diabetic is macro and microvascular complications as neuropathy. This is due to non-enzymatic protein glycation in vital organs forming nonfunctional proteins called advanced glycated products (AGEs). The highest levels of AGEs lead to effects that are more dangerous as neuropathy, cardiomyopathy, neuropathy and nephropathy 11. Consequently, the objective of this study is to explore the potential of a natural product to prevent or at least alleviate diabetic complications, with a focus on inhibiting the formation of advanced glycation products (AGEs) in the kidneys. The rational of current study to evaluate the potential of anserine combined with vitamin E in prevention of nephropathy in diabetic rats via inhibition of AGEs formation.

## Materials and Methods

### Animals

The handling with animals in this study was done according to ethical committee of King Abdulaziz University, Jeddah. This study included 60 male albino rats obtained from King Fahd Medical Research center, Jeddah. Animals were kept at normal condition for adaption one week. The rats were sorted into five groups (12 animals each): Normal control (GPI) and other four groups injected with single dose 55 mg/kg (i.p) streptozocin for induction of diabetes. After confirmation of diabetic by measuring blood glucose (≥250 mg/dl). The rats were either untreated (GPII) or GPIII treated with 1mg/kg b.w of anserine (i.p, GPIV (10 IU/kg b.w) vitamin E (i.p), or GPV; a combination of anserine and vitamin E. The treatment were daily for 3 months.

### Methods

At the end of experiment, animals were fasted for 14 hours and blood was collected from heart after anesthesia with thiopental. HbA1c was measured in whole blood. Serum was separated for measurement of glucose, advanced glycated products (AGEs), malondialdhyde (MDA) and inflammatory markers (TNF-α, IL-6). Kidney tissue was collected, stored at -20°C for measurement of MDA, reduced glutathione levels, superoxide dismutase, and catalase activities. All kits were obtained from Bio diagnostic company, Egypt.

### Statistical Analysis

The data was statistically analyzed using SPSS version 20.0, with significance at p value < 0.05. ANOVA one way test was used for comparison among groups. Correlation study between different groups.

## Results

**Fig 1:**
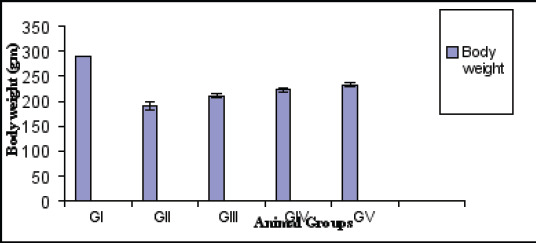
Body weight of animals at the end of experiment (Mean ±SD)

**Fig 2:**
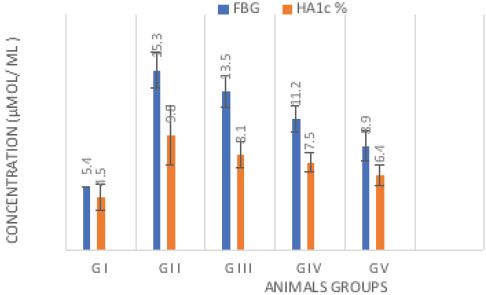
Fasting blood glucose and glycated hemoglobin (Mean ±SD)

**Fig 3:**
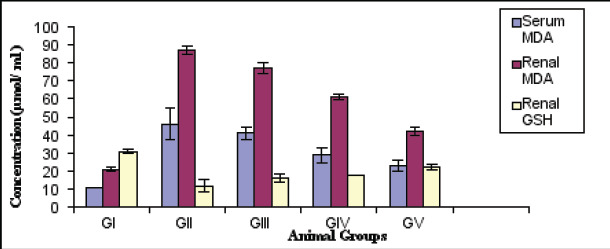
Oxidative stress markers of all studied groups (Mean ±SD)

Statistical analysis using ANOVA test showed a significant reduction in the body weight (gm) of diabetic rats versus control (p<0.001). However, diabetic treated with anserine or tocopherol alone or mixed showed a slight elevation in body weight compared with diabetic untreated (p<0.05).The combined effect is better than individual one (fig 1). Blood glucose and glycated hemoglobin levels were significantly elevated in diabetic rats versus normal (p<0.001) respectively. However, combined treatment with anserine and tocopherol lowered their levels compared with untreated on individual treatment one (p<0.001) (Fig 2). Additionally, combined treatment demonstrated improvement of oxidative stress biomarkers as MDA, GSH, SOD than individual one and untreated diabetic (p<0.001). In kidney tissue, GSH, catalase, and SOD levels were significantly reduced, while MDA levels were significantly increased in diabetic animals (fig 3). Treatment with anserine, vitamin E, or both improved these parameters (p<0.05). Inflammatory mediators as TNF-α and IL-6 and advanced glycated end products (AGEs) were significantly elevated in diabetic rats compared with control (p<0.001) and lowered by combined treatment with anserine and/or vitamin E but not returned to normal levels.

## Discussion

Aging is normal physiological events occurred during lifetime^12^. This is a accompanied declined in biological functions. One of most complications due to diabetic is protein glycation that affect many tissues mediated non-enzymatic reaction of blood glucose with tissue protein^13^ and lead to nonfunctional tissue^14^. New researches focused on utilization of peptides in treatment of some diseases. The study highlights on the potential of dipeptide anserine (β-alanyl-L-histidine) alone or and vitamin E as functional foods to counteract the effects of diabetic complications as nephropathy. These compounds appear to possess a protection against damage to vital organs such as the kidneys, eyes, heart, and lungs^15^-^20^. The reduction of blood glucose and glycated hemoglobin is mediatd by increased insulin sensitivity or decreased free radicals and enhance insulin secretion. In addition, anserine combined with vitamin E may bind with blood glucose that mask binding tissue proteins and prevent AGEs formation^21^. Thus reducing glycation of protein in tissues like the kidney. It also indicated that, these compounds can reduce the release of inflammatory mediators in renal cells, delaying the onset of diabetic nephropathy. The inflammatory mediators (IL-2 and TNF-α) contributed in inflammation of many tissues and affect its biological function 22. Their action on glycation (AGEs) is linked to their ability to suppress the release of free radicals, inhibit oxidation, and enhance antioxidant capacity. Importantly, they appear to be non-toxic for human use. Anserine may be effective due to its strong affinity for glucose, inhibiting glycation in kidney tissue 23. Meanwhile, α-tocopherol is known for its potent antioxidant properties, inhibiting peroxidation and AGEs formation.

Furthermore, these compounds reduce the overproduction of free radicals in diabetic rats, indicating their potential in inhibiting AGEs 24. This also indirectly protects against the harmful effects of AGEs. The study shows that they modulate the expression of inflammatory mediators, which are implicated in diabetic nephropathy^25^-^26^. Anserine and α-tocopherol were found to reduce aldose reductase, which converts fructose to sorbitol, and possess beneficial cell biological effects by lowering hyperglycemia-induced free radical production. In conclusion, these findings suggested that anserine and vit E may serve as a promising complementary and alternative therapy for various diabetic complications as nephropathy.

## Recommendation

It is recommended to increase awareness of supplementation natural products as regular dietary intake to avoid disease complications or delay the progress of metabolic disease as diabetic under medical supervision.

## Limitation of study

Studying the signaling pathway including kinases (JAK-STAT, PI3K-Akt, MAPK), not available due to cost and delay of shipment kits.

## Figures and Tables

**Figure 4:**
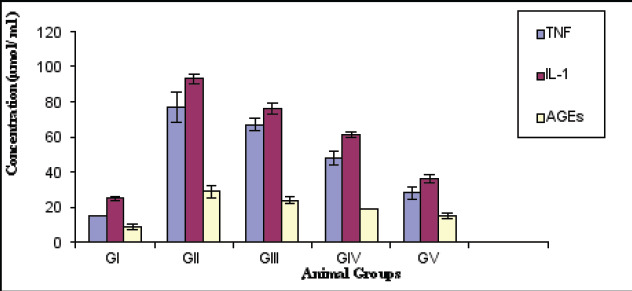
Serum TNF-α, IL-1 and AGEs levels in all studied groups (Mean+SD)
